# Double jeopardy from the COVID-19 pandemic: risk of exposure and income loss in Portugal

**DOI:** 10.1186/s12939-021-01569-1

**Published:** 2021-10-20

**Authors:** Ana Gama, Joana Alves, Daniela Costa, Pedro A. Laires, Patrícia Soares, Ana Rita Pedro, Marta Moniz, Luísa Solinho, Carla Nunes, Sónia Dias

**Affiliations:** 1 NOVA National School of Public Health, Public Health Research Centre, Universidade NOVA de Lisboa, Avenida Padre Cruz, 1600-560 Lisbon, Portugal; 2 Comprehensive Health Research Centre (CHRC), Campo Mártires da Pátria 130, 1169-056 Lisbon, Portugal

**Keywords:** Socioeconomic disparities, Mental health, COVID-19 pandemic, Vulnerable populations

## Abstract

**Background:**

Increasing evidence indicates that the first wave of the COVID-19 pandemic had immediate health and social impact, disproportionately affecting certain socioeconomic groups. Assessing inequalities in risk of exposure and in adversities faced during the pandemic is critical to inform targeted actions that effectively prevent disproportionate spread and reduce social and health inequities. This study examines i) the socioeconomic and mental health characteristics of individuals working in the workplace, thus at increased risk of COVID-19 exposure, and ii) individual income losses resulting from the pandemic across socioeconomic subgroups of a working population, during the first confinement in Portugal.

**Methods:**

This study uses data from ‘COVID-19 Barometer: Social Opinion’, a community-based online survey in Portugal. The sample for analysis comprised *n* = 129,078 workers. Logistic regressions were performed to estimate the adjusted odds ratios (AOR) of factors associated with working in the workplace during the confinement period and with having lost income due to the pandemic.

**Results:**

Over a third of the participants reported working in the workplace during the first confinement. This was more likely among those with lower income [AOR = 2.93 (2.64-3.25)], lower education [AOR = 3.17 (3.04-3.30)] and working as employee [AOR = 1.09 (1.04-1.15)]. Working in the workplace was positively associated with frequent feelings of agitation, anxiety or sadness [AOR = 1.14 (1.09-1.20)] and perception of high risk of infection [AOR = 11.06 (10.53-11.61)]. About 43% of the respondents reported having lost income due to the pandemic. The economic consequences affected greatly the groups at increased risk of COVID-19 exposure, namely those with lower education [AOR = 1.36 (1.19-1.56)] and lower income [AOR = 3.13 (2.47-3.96)].

**Conclusions:**

The social gradient in risk of exposure and in economic impact of the pandemic can result in an accumulated vulnerability for socioeconomic deprived populations. The COVID-19 pandemic seems to have a double effect in these groups, contributing to heightened disparities and poor health outcomes, including in mental health. Protecting the most vulnerable populations is key to prevent the spread of the disease and mitigate the deepening of social and health disparities. Action is needed to develop policies and more extensive measures for reducing disproportionate experiences of adversity from the COVID-19 pandemic among most vulnerable populations.

## Introduction

The socioeconomic inequalities in health are a global phenomenon. People in a socially disadvantaged position are generally more likely to get sick, have worse life expectancy and higher mortality rates compared with more advantaged socioeconomic groups [1]. However, not only the people who are poor tend to have worse health outcomes than rich, but also, the lower people are positioned in the social hierarchy, the greater are these differences. This so-called social gradient in health has been long described across a range of diseases, and this is likely no exception regarding COVID-19 [2, 3].

The first outbreak of COVID-19, an infectious disease caused by the severe acute respiratory syndrome coronavirus 2 (SARS-CoV-2), was reported in Wuhan, China, in December 2019. The World Health Organization declared COVID-19 a global public health emergency and classified it as a pandemic in March 2020. In many countries worldwide, including Portugal, mitigation strategies were implemented in the following months, comprising imposition of mobility restrictions, social distancing, use of mask and handwashing, isolation (in case of infection) and lockdown. Generally, public spaces and non-essential services and establishments were closed. In Portugal, the first period of confinement and lockdown lasted over 7 weeks, after which restrictions started to be lifted gradually and differentially across sectors and regions.

Increasing evidence suggests that the first wave of the COVID-19 pandemic had immediate health and social impact that disproportionately affected certain socioeconomic groups [4, 5]. In order to further understand the impact of the pandemic in societies, a social determinants of health approach has been advocated by several academics, highlighting the role of key structural determinants in contributing to disproportionate effects on most vulnerable groups [6–8]. Structural drivers of inequities such as adverse working conditions have increased health risks and enabled inequitable distribution of income [7]. A significant proportion of COVID-19 cases was found among individuals with occupations that require close contact and interaction with colleagues and the general public, such as retail staff, cleaners, healthcare professionals and cruise ship crews [9, 10]. Low education levels, associated with low health literacy, may hinder the capacity for adopting the recommended preventive measures to reduce the spread of the disease. In addition, low education is one of the most important determinants of employment status, with a significant impact on income level [11, 12]. According to OECD reports, adults with low education across Europe and the USA are at increased risk for unemployment compared with their better educated counterparts [13]. Once in the workforce, low-educated and low-skilled workers face a higher risk of holding a temporary contract [14–16]. The precarious working conditions might hinder social distancing and make people dependent on temporary jobs, ruling out the possibility of confinement. Additionally, people with low income are more likely to live in disadvantaged neighbourhoods in crowded housing, limiting the possibilities for proper distancing and adequate sanitation [17]. Research has found that people living in more deprived and higher density populated areas were more likely to test positive for COVID-19 [18].

Not only has the COVID-19 pandemic revealed persisting social inequalities in populations health, but it may also be widening the existing disparities. It has been argued that there is an unequal impact of COVID-19 across the population, affecting more heavily those who live in deprivation [2–4]. An online survey in the UK showed that people with lower socioeconomic position were more likely to experience income cuts and job loss during the early weeks of lockdown. This group was also more likely to be unable to make ends meet and to access adequate food and required medication [19].

In addition, evidence has highlighted the relationship between the pandemic and mental health, with self-reported anxiety and stress being common psychological reactions during the COVID-19 crisis [5], which may affect disproportionately socioeconomic disadvantaged groups.

Although the literature suggests that there is likely a social gradient in COVID-19, studies examining inequalities in risk of exposure and in the adversities faced during the pandemic with data on individual socioeconomic indicators such as occupation, income and education are still scarce, particularly for Portugal. Evidence on the role of social inequalities as both a risk factor for infection and an outcome of the pandemic is critical to assist in developing targeted interventions that effectively prevent disproportionate spread and reduce social and health inequities.

This study aimed to examine i) the socioeconomic and mental health characteristics of individuals working in the workplace, thus at increased risk of COVID-19 exposure, and ii) individual income losses resulting from the pandemic across socioeconomic subgroups of workers, during the first confinement in Portugal.

## Methods

This study draws from ‘COVID-19 Barometer: Social Opinion’, a community-based survey to assess individuals perceived health status (including mental health), socioeconomic conditions, health care utilisation and adherence to mitigation measures during the COVID-19 pandemic. It is an ongoing open cohort study that started on March 2020, where individuals aged ≥16 years old may fill the online questionnaire once or regularly over time. The questionnaire is flexible to adjust rapidly to the dynamic characteristics of the pandemic. The methods and procedures used are described elsewhere [20–22].

This cross-sectional study used the latest available response from each participant obtained since March 26th 2020 (1 week after legal enforcement of lockdown began) and during the following 9 weeks (until May 22nd 2020). The study included only participants who reported working as their current activity. The final sample considered for analysis comprised *n* = 129,078 respondents.

### Variables

Two main outcomes were examined. The outcome variable related to risk of exposure was working in the workplace during the confinement period. Participants were asked about how they were developing their professional activity. The response options were ‘Teleworking’, ‘Working in the workplace, with contact with colleagues or the public’, ‘Working in the workplace, without contact’, ‘Suspended activity’ and ‘Not applicable’. For the analysis, the dichotomic variable “Work arrangements” was created with the categories ‘Working in the workplace’ (grouping ‘in contact with colleagues or the public’ and ‘without contact’ options) and ‘Teleworking’. The outcome variable related to the economic impact of the pandemic was individual income loss. Participants were asked whether they had lost income due to the pandemic, with response options being ‘No’, ‘Yes, lost partial income’ and ‘Yes, lost total income; for the analysis, these response options were recategorized into ‘No’ and ‘Yes’ (grouping ‘partial’ and ‘total’ income lost). The question about income loss was added during the study period, on April 11th 2020 (about 1 month after legal enforcement of lockdown started).

Sociodemographic characteristics were assessed as independent variables, including sex, age, education level and monthly household income (including salaries and other sources of income such as subsidies, rent, monetary support, alimony). Participants were also asked about their current activity, with the response options being ‘working as an employee’, ‘working as self-employed’, ‘unemployed’, ‘student’, ‘housekeeper’, ‘retired’ and ‘other’; for the analysis, only the options ‘employee’ and ‘self-employed’ were considered.

Participants were also asked about how frequently they felt agitated, anxious, or sad due to the pandemic mitigation measures, with the options being ‘Everyday’, ‘Almost all days’, ‘Some days’ and ‘Never’, and about their self-perceived risk to get COVID-19 infection – ‘high’, ‘moderate’, low’ and ‘no risk’.

### Data analysis

Descriptive analyses were performed using Chi-square test to examine independence between sociodemographic characteristics, frequency of feeling agitated, anxious, or sad, COVID-19 risk perception and the outcome variables. Logistic regression analyses were performed to estimate the crude odds ratios (OR) and adjusted odds ratios (AOR), with 95% confidence intervals (CI), of factors associated with working in the workplace during the confinement period and with having lost income due to the pandemic. The models were adjusted for all independent variables. Data analysis was performed using IBM SPSS Statistics v.26.

### Ethical approval

The survey was approved by the Ethics Commission of the NOVA National School of Public Health (Ref: CE/ENSP/CREE/2/2020). Anonymity of participants and confidentiality of data were guaranteed. Informed consent was obtained from all participants. The database is stored in device protected by password, with exclusive access of the research team.

## Results

The sample characteristics are presented in Table 1. Overall, 64.2% of the participants were women and 60.6% were between 16 and 45 years old. In total, 29.1% of the sample had basic or secondary education, 22.5% reported a monthly household income up to 1000€ and 23.6% 1001-1500€. The majority of the participants were employee (80.4%) and 19.6% were self-employed. Over a half of participants (55.4%) reported feeling agitated, anxious or sad some days, but 25.8% reported having these feelings every day or almost all days. Overall, 44.8% perceived to have a moderate risk of infection and 33.2% to have low or no risk, but 22.0% perceived high risk.

**Table 1:** Characteristics of the participants

	Total (***n*** = 129,078)
	n (%)
**Sex (*n*** **= 128,989)**	
Women	82,771 (64.2)
Men	46,218 (35.8)
**Age (n = 129,078)**	
16-25	5682 (4.4)
26-45	72,580 (56.2)
> 45	50,816 (39.4)
**Education level (n = 128,752)**	
Basic education	6292 (4.9)
Secondary education	31,135 (24.2)
Higher education	91,325 (70.9)
**Monthly household income (*n*** **= 117,836)**	
< 650€	6329 (5.4)
651-1000€	20,134 (17.1)
1001-1500€	27,773 (23.6)
1501-2000€	22,897 (19.4)
2001-2500€	17,163 (14.6)
> 2500€	23,540 (20.0)
**Employment status (n = 129,078)**	
Employee	103,725 (80.4)
Self-employed	25,353 (19.6)
**Frequency of feeling agitated, anxious or sad (n = 128,459)**	
Every day/almost all days	33,213 (25.8)
Some days	71,127 (55.4)
Never	24,119 (18.8)
**COVID-19 risk perception (*n*** **= 115,427)**	
High risk	25,392 (22.0)
Moderate risk	51,716 (44.8)
Low/no risk	38,319 (33.2)

The analysis of the work arrangements showed that 34.8% of the participants were working in the workplace (Table 2). These participants were more frequently male, were younger, had lower education and lower household income level, and reported more often working as employee than participants who were teleworking. A higher proportion of participants working in the workplace reported frequent feelings of agitation, anxiety or sadness and perceived high risk of infection, compared with participants teleworking.

**Table 2:** Characteristics of the participants by work arrangements and factors associated with working in the workplace

	Work arrangements			Working in the workplace			
	Working in the workplace n (%)	Teleworking n (%)	***p-value***	Crude OR (CI 95%)	***p-value***	Adjusted OR (CI 95%)	***p-value***
**Total**	32,990 (34.8)	61,872 (65.2)					
**Sex (*n*** **= 94,803)**							
Women	19,246 (58.4)	40,281 (65.1)	*< 0.001*	1		1	
Men	13,722 (41.6)	21,554 (34.9)		1.33 (1.30-1.37)	*< 0.001*	1.20 (1.16-1.24)	*< 0.001*
**Age (n = 94,862)**							
16-25	1790 (5.4)	1937 (3.1)	*< 0.001*	1.96 (1.83-2.10)	*< 0.001*	1.73 (1.58-1.88)	*< 0.001*
26-45	18,586 (56.4)	33,181 (53.6)		1.19 (1.16-1.22)	*< 0.001*	1.16 (1.12-1.20)	*< 0.001*
> 45	12,614 (38.2)	26,754 (43.3)		1		1	
**Education level (n = 94,666)**							
Basic/Secondary education	12,967 (39.4)	9245 (15.0)	*< 0.001*	3.69 (3.58-3.81)	*< 0.001*	3.17 (3.04-3.30)	*< 0.001*
Higher education	19,935 (60.6)	52,519 (85.0)		1		1	
**Monthly household income (*n*** **= 87,185)**							
< 650€	1839 (6.0)	1034 (1.8)	*< 0.001*	5.10 (4.70-5.54)	*< 0.001*	2.93 (2.64-3.25)	*< 0.001*
651-1000€	6212 (20.3)	6494 (11.5)		2.75 (2.62-2.88)	*< 0.001*	1.80 (1.70-1.91)	*< 0.001*
1001-1500€	7655 (25.0)	12,531 (22.1)		1.75 (1.68-1.83)	*< 0.001*	1.38 (1.32-1.45)	*< 0.001*
1501-2000€	5611 (18.4)	11,720 (20.7)		1.37 (1.31-1.44)	*< 0.001*	1.18 (1.12-1.24)	*< 0.001*
2001-2500€	4062 (13.3)	9961 (17.6)		1.17 (1.11-1.23)	*< 0.001*	1.11 (1.05-1.17)	*< 0.001*
> 2500€	5186 (17.0)	14,880 (26.3)		1		1	
**Employment status (n = 94,862)**							
Employee	29,137 (88.3)	53,277 (86.1)	*< 0.001*	1.22 (1.17-1.27)	*< 0.001*	1.09 (1.04-1.15)	*< 0.001*
Self-employed	3853 (11.7)	8595 (13.9)		1		1	
**Frequency of feeling agitated, anxious or sad (n = 94,447)**							
Every day/almost all days	9896 (30.2)	13,577 (22.0)	*< 0.001*	1.54 (1.48-1.61)	*< 0.001*	1.14 (1.09-1.20)	*< 0.001*
Some days	16,885 (51.6)	35,517 (57.6)		1.01 (0.97-1.04)	*0.723*	0.93 (0.89-0.97)	*0.001*
Never	5958 (18.2)	12,614 (20.4)		1		1	
**COVID-19 risk perception (*n*** **= 85,902)**							
High risk	13,306 (43.6)	7195 (13.0)	*< 0.001*	10.36 (9.91-10.83)	*< 0.001*	11.06 (10.53-11.61)	*< 0.001*
Moderate risk	13,231 (43.3)	25,787 (46.6)		2.87 (2.76-2.99)	*< 0.001*	3.06 (2.93-3.19)	*< 0.001*
Low/no risk	3996 (13.1)	22,387 (40.4)		1		1	

The logistic regression analysis confirmed that the odds of working in the workplace were higher among men [AOR = 1.20 (1.16-1.24)], younger participants [16-25 years: AOR = 1.73 (1.58-1.88); 26-45 years: AOR = 1.16 (1.12-1.20), compared with > 45 years age group], lower education [basic or secondary education [AOR = 3.17 (3.04-3.30), compared with higher education] and increased with decreasing income level [< 650€: AOR = 2.93 (2.64-3.25), compared with > 2500€]. Odds of working in the workplace were also higher among employee participants [AOR = 1.09 (1.04-1.15)], as well as those feeling agitated, anxious or sad every day or almost all days [AOR = 1.14 (1.09-1.20), compared with never] and those perceiving high risk of infection [AOR = 11.06 (10.53-11.61), compared with low/no risk].

The analyses of income loss showed that 42.9% of the respondents lost income due to the pandemic (Table 3). These participants were younger, had lower education and lower household income levels, and reported more frequently being self-employed than those who did not lose income. Reported income loss was also more common among participants reporting higher frequency of feelings of agitation, anxiety or sadness.

**Table 3:** Characteristics of the participants by reported income loss and factors associated with having lost income

	Total (***n*** = 6477)	Having lost income due to the pandemic			Income loss			
		Yes n (%)	No n (%)	***p-value***	Crude OR (CI 95%)	***p-value***	Adjusted OR (CI 95%)	***p-value***
**Total**		2776 (42.9)	3701 (57.1)					
**Sex (*n*** **= 6471)**								
Women	4482 (69.3)	1936 (69.7)	2546 (68.9)	0.470	1.04 (0.94-1.16)	*0.470*	0.92 (0.81-1.05)	*0.202*
Men	1989 (30.7)	840 (30.3)	1149 (31.1)		1		1	
**Age (n = 6477)**								
16-25	1484 (22.9)	800 (28.8)	684 (18.5)	< 0.001	2.61 (2.26-3.01)	*< 0.001*	1.74 (1.45-2.09)	*< 0.001*
26-45	3168 (48.9)	1411 (50.8)	1757 (47.5)		1.79 (1.59-2.02)	*< 0.001*	1.62 (1.40-1.87)	*< 0.001*
> 45	1825 (28.2)	565 (20.4)	1260 (34.0)		1		1	
**Education level (*n*** **= 6442)**								
Basic/Secondary education	2774 (43.1)	1432 (51.8)	1342 (36.5)	< 0.001	1.87 (1.69-2.07)	*< 0.001*	1.36 (1.19-1.56)	*< 0.001*
Higher education	3668 (56.9)	1331 (48.2)	2337 (63.5)		1		1	
**Monthly household income (*n*** **= 5914)**								
< 650€	858 (13.5)	548 (21.7)	310 (9.1)	< 0.001	4.65 (3.83-5.64)	*< 0.001*	3.13 (2.47-3.96)	*< 0.001*
651-1000€	1308 (22.1)	681 (26.9)	627 (18.5)		2.85 (2.40-3.39)	*< 0.001*	2.16 (1.76-2.65)	*< 0.001*
1001-1500€	1210 (20.5)	502 (19.9)	708 (20.9)		1.86 (1.56-2.22)	*< 0.001*	1.57 (1.29-1.91)	*< 0.001*
1501-2000€	876 (14.8)	321 (12.7)	555 (16.4)		1.52 (1.25-1.84)	*< 0.001*	1.34 (1.09-1.65)	*0.006*
2001-2500€	610 (10.3)	184 (7.3)	426 (12.6)		1.13 (0.91-1.41)	*0.258*	1.11 (0.88-1.41)	*0.365*
> 2500€	1052 (17.8)	290 (11.5)	762 (22.5)		1		1	
**Employment status (n = 6477)**								
Employee	5461 (84.3)	2016 (72.6)	3445 (93.1)	< 0.001	1		1	
Self-employed	1016 (15.7)	760 (27.4)	256 (6.9)		5.07 (4.36-5.90)	*< 0.001*	5.96 (5.01-7.08)	*< 0.001*
**Frequency of feeling agitated, anxious or sad (*n*** **= 6445)**								
Every day/almost all days	1462 (22.7)	762 (27.6)	700 (19.0)	< 0.001	2.52 (2.13-2.98)	*< 0.001*	2.02 (1.66-2.46)	*< 0.001*
Some days	3969 (61.6)	1694 (61.3)	2275 (61.8)		1.72 (1.49-2.00)	*< 0.001*	1.70 (1.43-2.02)	*< 0.001*
Never	1014 (15.7)	306 (11.1)	708 (19.2)		1		1	

The logistic regression analysis showed that the likelihood of having lost income was higher among the youngest participants [AOR = 1.74 (1.45-2.09), compared with those aged > 45 years old], those with lower education [basic or secondary education: AOR = 1.36 (1.19-1.56), compared with higher education], those with lower household income levels [< 650€: AOR = 3.13 (2.47-3.96), compared with > 2500€] and those self-employed [AOR = 5.96 (5.01-7.08), compared with employed individuals]. In addition, participants who reported feeling agitated, anxious or sad every day or almost all days were twice as likely to have lost income [AOR = 2.02 (1.66-2.46)] than individuals that never had those feelings.

## Discussion

This study draws from a community-based online survey that collects individual data on socioeconomic and health indicators during the COVID-19 pandemic, and sheds light on disparities in both risk of exposure and impact of the pandemic in a sample of working population in Portugal.

Over a third of the participants reported working in the workplace during the first period of confinement, and this was more likely among those with lower income, lower education and working as employee. These findings are in line with the literature suggesting that people from lower socioeconomic backgrounds have less ability to be resilient to shocks, such as pandemics and their effects [23]. Low-income workers are more likely to have a precarious financial situation, low savings, limited opportunity to access to paid sick leave, and face strong disincentives to be absent from work because they cannot afford to lose money [24, 25]. Employees, in particular, often lack the power to choose to stay home in the event of an outbreak [26]. In the context of COVID-19 crisis, these circumstances contribute for an increased risk of exposure among these groups. Professional activities from low social strata usually have adverse working conditions, including long or irregular working hours and shift work, where often remote work is not possible (e.g., retail, cleaning) [27]. In addition, many low-wage workers do not have a personal vehicle, relying on public transportation to travel to their workplaces, where large crowds cannot always be avoided, and social distancing is difficult to ensure. This is consistent with the perception of the high risk of infection found among the participants working in the workplace, compared with those teleworking. Low education can also play an important role in risk exposure. In addition to the well-documented link of low education to low-wage jobs in areas where work cannot be performed remotely, low education has been associated with lower health literacy skills [28–30]. Effective public health communication to adopt the recommended preventive measures during an infectious disease outbreak depends on people being able to access and understand the information. Individuals with limited health literacy may be more easily misguided by incorrect sources of information, misperceive risks and overlook precautions, and have less ability to comply with preventive measures [31, 32], despite likely being at increased risk of exposure.

Our results show that the economic consequences of the pandemic were felt at a greater extent by the same groups at increased risk of exposure, with income loss being more likely among those with lower education and lower income. Indeed, emerging literature indicates that the economic impact of the pandemic is being disproportionately shouldered by the populations with poor socioeconomic background [33, 34]. For instance, lower education groups frequently are more likely to hold temporary and/or precarious job contracts [35, 36]. Our findings show that the economic impact of the pandemic has also been significantly worse on the youngest workers. Previous data indicate that the youth were already vulnerable within the workforce prior to this crisis given the growing precarious work, suggesting that the recovery from this shock will be more difficult and take more time for this group [37, 38]. Our findings also indicate that self-employed individuals faced higher income loss. It is plausible that the working conditions, including income, of sole business owners are more unstable than those of paid employees. Competition may be harder for sole proprietors, making them dependent on a few main customers, which can result in business closures in a context of lockdown.

This study shows that feelings of agitation, anxiety and sadness due to the pandemic are associated with both risk of exposure by working in the workplace and the economic impact of the pandemic in terms of income loss. The findings indicate that more psychologically vulnerable individuals are more likely to have jobs where remote work is not possible, which has been associated with professional activities from low social strata, lower socioeconomic position and with higher job insecurity [27]. In our study, working in the workplace was also associated with the perception of high risk of becoming infected, so we hypothesize that the risk of exposure might also make respondents become more afraid of being infected, and thus, more anxious. Indeed, research has shown that employees working on essential activities that cannot be performed from home (e.g. security forces, cleaning, healthcare) have experienced increased feelings of helplessness to external threats [39]. In addition, the findings indicate that individuals reporting frequent psychological symptoms such as stress and anxiety had greater income losses. One can speculate that the mental health condition of these individuals might have implications on their capacity to work, potentially resulting in suspension of activity, medical leave and losing income. But the possibility of reverse causation cannot be excluded. In fact, studies conducted during the COVID-19 pandemic and in prior crises reported poor mental health outcomes such as post-traumatic stress disorder, depression, psychological distress, insomnia and stress [40–43]. This may be the case for people working as self-employed, including in the shadow economy and informal activities, which most of the times are low paid, lack social protection and increase exposure to psychosocial stressors (e.g., highest financial insecurity, fear of job loss or not being able to make ends meet) [44, 45]. Increasing evidence suggests that a mental health epidemic is occurring along with the COVID-19 pandemic, which demands the attention of the global health community [41, 43].

It is worthy to mention that young men with low education experienced a higher risk of COVID-19 exposure due to working in the workplace, compared to women. This may be explained by the fact that, though female workers in many European countries as in Portugal account for the majority of health and social care workers (so called essential workers) whose activities are performed in the workplace [46], it is male workers who predominantly perform many other essential activities that cannot be executed remotely and do not demand high qualification (e.g. security forces, waste collection, transportation). In addition, there is evidence that during the first lockdown, with the closure of schools and the increased care needs among older people, the demand for informal care provision fell more heavily on women [47]. Women were estimated to be doing three quarters of the unpaid care work, and this has increased during the COVID-19 pandemic [7, 47]. In our study, no gender differences were found in terms of income loss. However, this finding cannot be overlooked, as medium and long-term effects of the pandemic on the careers of female workers in many sectors are beginning to be assessed [48, 49].

Based on this study’s findings, it is fair to consider that the COVID-19 pandemic, by worsening pre-existent inequalities, has soon contributed to enlarge the gap between the socioeconomic strata in societies. The social gradient in risk of exposure and in economic impact of the pandemic, along with the combination of these two circumstances, can result in an accumulated vulnerability for socioeconomic deprived populations. The COVID-19 pandemic seems to have a double effect in these groups, contributing to heightened disparities and poor health outcomes, including in mental health.

Limitations of this study must be acknowledged. The sample is not representative. The online format of the survey favoured the participation of individuals with access to digital technologies and digitally literate, such as those with higher education. Also, the survey may have been subjected to volunteer bias (e.g. more engaged and informed citizens completed the survey). The study was based on the analysis of self-reported data, which may have resulted in recall bias. There is also a potential risk of social desirability bias, nevertheless the anonymous online nature of this survey may have helped to minimise this impact. As the data are drawn from a cross-sectional survey, any inferences about causality are not possible. Specific information on the nature of occupation or job task of participants was not available. This study draws from an ongoing survey in Portugal, making one of the largest community-based surveys performed on COVID-19 so far, with data collected on how individuals perceive, act and live during the pandemic, and which health, social and economic effects they have experienced during the confinement and deconfinement periods.

## Conclusions

This study indicates that the COVID-19 pandemic has reinforced the broad socioeconomic and health disparities within our society. Protecting the most vulnerable populations is key to prevent the spread of the disease and mitigate the deepening of disparities. Action is needed to develop policies and more extensive measures for reducing disproportionate experiences of adversity from the COVID-19 pandemic among most vulnerable populations. Additionally, further attention is needed on planning for future pandemics, including the creation and strengthening of mechanisms of social, economic and health support that increase populations capacity to cope and recover from similar crises, including the youngest and the most socially disadvantaged subgroups. Social determinants of health must continue to be addressed in public health research, policy and action.

## Data Availability

The dataset used and analysed during the current study is available from the corresponding author on reasonable request. The data are not publicly available since this is an ongoing study.
